# Influence of soil minerals on chromium(VI) reduction by sulfide under anoxic conditions

**DOI:** 10.1186/1467-4866-8-4

**Published:** 2007-04-12

**Authors:** Yeqing Lan, Baolin Deng, Chulsung Kim, Edward C Thornton

**Affiliations:** 1 College of Sciences, Nanjing Agricultural University, Nanjing, 210095, China; 2 Dept. of Civil and Environmental Engineering, University of Missouri-Columbia, Columbia, MO 65211, USA; 3 Dept. Environmental Science, University of Dubuque, Dubuque, IA 52001, USA; 4 Field Hydrology and Chemistry Group, Pacific Northwest National Laboratory (PNNL), Richland, Washington 99352, USA

## Abstract

The effects of soil minerals on chromate (Cr^VI^O_4_^2-^, noted as Cr(VI)) reduction by sulfide were investigated in the pH range of 7.67 to 9.07 under the anoxic condition. The examined minerals included montmorillonite (Swy-2), illite (IMt-2), kaolinite (KGa-2), aluminum oxide (γ-Al_2_O_3_), titanium oxide (TiO_2_, P-25, primarily anatase), and silica (SiO_2_). Based on their effects on Cr(VI) reduction, these minerals were categorized into three groups: (i) minerals catalyzing Cr(VI) reduction – illite; (ii) minerals with no effect – Al_2_O_3_; and (iii) minerals inhibiting Cr(VI) reduction- kaolinite, montmorillonite, SiO_2 _and TiO_2 _. The catalysis of illite was attributed primarily to the low concentration of iron solubilized from the mineral, which could accelerate Cr(VI) reduction by shuttling electrons from sulfide to Cr(VI). Additionally, elemental sulfur produced as the primary product of sulfide oxidation could further catalyze Cr(VI) reduction in the heterogeneous system. Previous studies have shown that adsorption of sulfide onto elemental sulfur nanoparticles could greatly increase sulfide reactivity towards Cr(VI) reduction. Consequently, the observed rate constant, *k*_obs_, increased with increasing amounts of both iron solubilized from illite and elemental sulfur produced during the reaction. The catalysis of iron, however, was found to be blocked by phenanthroline, a strong complexing agent for ferrous iron. In this case, the overall reaction rate at the initial stage of reaction was pseudo first order with respect to Cr(VI), i.e., the reaction kinetics was similar to that in the homogeneous system, because elemental sulfur exerted no effect at the initial stage prior to accumulation of elemental sulfur nanoparticles. In the suspension of kaolinite, which belonged to group (iii), an inhibitive effect to Cr(VI) reduction was observed and subsequently examined in more details. The inhibition was due to the sorption of elemental sulfur onto kaolinite, which reduced or completely eliminated the catalytic effect of elemental sulfur, depending on kaolinite concentration. This was consistent with the observation that the catalysis of externally added elemental sulfur (50 μM) on Cr(VI) reduction would disappear with a kaolinite concentration of more than 5.0 g/L. In kaolinite suspension, the overall reaction rate law was:

-d[Cr(VI)]/dt = *k*_obs_[H^+^]^2^[Cr(VI)][HS^-^]^0.70^

## Background

Chromium (Cr) pollution is widely concerned throughout the world because of its large magnitude and known adverse health effect [1]. For example, dregs of chromium are produced during manufacturing Cr-containing alloys and salts. These waste materials usually contain a significantly amount of chromium [2], which could be leached out due to water filtration and/or ground water fluctuation. Chromium contamination of soils and water is also caused by improper disposals of Cr-containing waste water and sludges as used for corrosion inhibition and electroplating industries.

When released into the environment, chromium exists mainly as hexavalent chromate (HCrO_4_^-^/CrO_4_^2-^, noted as Cr(VI)) and trivalent forms (noted as Cr(III)). Chromium species at different oxidation states show substantially different physical and chemical properties. Cr(VI) is an oxidant with high solubility and mobility in soils and aquifers, as well as high toxicity to human and ecosystems; whereas Cr(III) has lower mobility, mostly precipitated as hydroxides or adsorbed onto mineral surfaces. As a result, contamination of Cr(VI) is often controlled by Cr(VI) reduction using various reductants, such as zero valent iron[3], ferrous ions [4-11], and organic compounds [12-14]. Most of these studies aimed to reduce Cr(VI) in the aqueous phase relevant to groundwater remediation by delivering reductants in solid or liquid forms.

For contamination residing in the vadose-zone, mixing a reactive agent in solid form is often difficult to manipulate and using a liquid reductant runs the risk of further spreading the contaminants to previously uncontaminated zones. A new technology, *in situ *gaseous reduction (ISGR), has therefore been developed by Pacific Northwest National Laboratory by using gaseous hydrogen sulfide (H_2_S) as the reductant[15,16]. Laboratory experiments showed that over 90% of Cr(VI) could be reduced to Cr(III) in the soils by H_2_S treatment [16]. A field study at the White Sand Missile Range, New Mexico, USA, also demonstrated over 70% of Cr immobilization through its reduction to Cr(III) [15]. The field demonstration further showed that hydrogen sulfide could be handled safely for field application [17]. Secondary contamination of H_2_S is not taking place because the residual gas could be consumed by reaction with iron oxides in soils[18]. The ISGR approach has advantages of easy controls of the reductant delivery and cost-effectiveness.

To design and optimize the ISGR system for reductive Cr(VI) immobilization in soils, the reaction kinetics between Cr(VI) and H_2_S in heterogeneous systems must be fully characterized. Kinetics and mechanism of Cr(VI) reduction by H_2_S in the aqueous phase have been established [19,20]. The overall reaction was second order, i.e., first order with respect to Cr(VI) and first order to sulfide. Elemental sulfur was the major product of sulfide oxidation under the anaerobic condition. Lan et al. [21] further demonstrated that elemental sulfur nanoparticles could greatly catalyze Cr(VI) reduction by sulfide. These aqueous phase processes are relevant to ISGR treatment in the vadose zone because even though the treatment is by gaseous reductant, Cr(VI) reduction occur in the water film on mineral particle surfaces formed under suitable humidity [22].

Heterogeneous phase Cr(VI) reduction by other reductants were also widely studied. Eary and Rai [23] observed Cr(VI) reduction by hematite and biotite over a wide pH range, and proposed that the dissolution of ferrous iron from solid phases should take place prior to Cr(VI) reduction, i.e., the reduction occurs in the solution phase rather than at surface sites. Patterson and Fendorf [24] demonstrated that freshly prepared ferrous sulfide reduced Cr(VI) quite effectively throughout the pH range of 5.0 to 8.0 and reaction occurred at solid-solution interface. Buerge and Hug[25] showed that the rate of Cr(VI) reduction by ferrous iron was significantly increased by several minerals including goethite (α-FeOOH), lepidocrocite (γ-FeOOH), montmorillonite (SAz-1), kaolinite (China Clay), and amorphous silica (Aerosil OX50), but not by aluminum oxide (Aluminiumoxid C). In another study, minerals, such as aluminum oxide (γ-Al_2_O_3_), goethite (α-FeOOH) and titanium dioxide (TiO_2_, anatase) were found to catalyze Cr(VI) reduction by α-hydroxyl carboxylic acids and their esters, α-carbonyl carboxylic acids, and substituted phenols [26,27].

Whether soil minerals will have a major impact on Cr(VI) reduction by sulfide has not been reported. As part of our effort to better understand the reaction between Cr(VI) and S^2- ^in soils, this study aims to: (1) investigate the overall effect of various minerals on the reduction of Cr(VI) by sulfide; (2) examine the role of Fe(III)/Fe(II) as a component of some minerals in catalyzing Cr(VI) reduction reaction, and (3) investigate the effect of element sulfur on the reaction in the heterogeneous system where elemental sulfur can be adsorbed on the surfaces of minerals. The examined minerals included montmorillonite, illite, kaolinite, aluminum oxide, anatase, and silica, all common soil minerals [28]. Initial reactant concentrations were set at micromolar level for chromate and millimolar level for sulfide to facilitate kinetic data collection, and were also within the concentration range found at the contamination sites [1]. The study was conducted in alkaline conditions with pH values from 7.7 to 9.1. The rationale was that the alkaline condition dominated at the Hanford site (Washington State, USA), where the application of ISGR technology was geared to. A recent study showed pH values ranging from 7,71 to 8.11 in some Hanford groundwater samples [29].

## Experimental methods

### Chemicals

Potassium dichromate, elemental sulfur (S_8_), diphenyl carbazide, and acetone were obtained from Sigma-Aldrich Company; and boric acid, sodium phosphate, sodium hydroxide, sodium sulfide (Na_2_S•9H_2_O), sulfuric acid, hydrogen chloride, ferrous chloride, and diammonium hydrogen phosphate were from Fisher Scientific. The chemicals were at least ACS reagent grade and used without further purification, except for sodium sulfide crystals that were rinsed with degassed Milli-Q water (with18.2 MΩ-cm resistivity, Millipore Corp.) to remove the oxidized surface layer. Stock solutions of chromate and sulfide were prepared by Milli-Q water purged thoroughly with high purity nitrogen gas and stored in amber bottles placed in an anaerobic chamber (Models 855-AC, PLAS-LABS, INC.) prior to use. Stock solution of elemental sulfur was prepared by dispersing 1.0 g elemental sulfur (S_8_) in 50 ml acetone. When measured amount of elemental sulfur stock solution was added to reaction system (water solution), acetone concentration was always less than 2% and sulfur colloid formed immediately due to the change in solubility. Glassware was cleaned by soaking in 1 M HCl for at least 3 hrs and then thoroughly rinsed by water and Milli-Q water.

### Minerals

Montmorillonite (Swy-2), illite (IMt-2), kaolinite (KGa-2) used in this study were obtained from the Source Clay Minerals Repository, University of Missouri-Columbia (U.S.A). Aluminum oxide (γ-Al_2_O_3_), silicon oxide (SiO_2_), and titanium oxide (TiO_2_, P-25, primarily anatase) were from Degussa Corporation. Point of zero charge (PZC) and BET specific surface area (SSA) of minerals were listed in Table 1.

**Table 1:** pH of Point of Zero Charge and BET specific surface area of tested minerals.

Minerals	pH_PZC_	SSA(m^2^/g)
Aluminum oxide	8.9^a^	90.1^a^
Silicon oxide	2.3^a^	90.0^a^
Titanium oxide	6.5^c^	40.5^a^
Montmorillonite	5.9^d^	99.0^d^
Kaolinite	4.5–5.0^b^	22.4^b^
Illite	3.5^b^	24.0^b^

Sources: a – Degussa (1991); b – this work; c – Torrents and Stone (1991); d – Buerge and Hug (1999)

### Experimental procedure

Experiments were mostly performed in an anaerobic chamber (N_2_, balanced by 10% H_2_) with a temperature of 24.0 ± 0.5°C, including experimental setup and chemical analyses. Solution pH was controlled by 0.10 M borate buffer. No strong electrolyte was used for ionic strength control in this study, since our preliminary experiments indicated that the reaction was independent of ionic strength when it was between 0.0 and 1.0 M.

Kinetic experiments examining the effect of various minerals on Cr(VI) reduction by sulfide at pH7.87 were started by purging an adequate amount of buffer solution in a 40 ml amber bottle with high purity nitrogen gas for 20 min. The vessel was closed immediately by a screw cap with Teflon/silicon septum to prevent air from getting into the solution again. Then, the buffer solution was transferred into the anaerobic chamber. A selected mineral (as dry power) and 0.80 ml of 1.00 mM K_2_Cr_2_O_7 _stock solution were added into the amber bottle and the suspension was mixed for 30 min. This allowed dispersion of mineral particles and hydration equilibration with the buffer solution. An adequate amount of sulfide stock solution was then introduced to initiate the reaction. The final total volume of the suspension was 40 ml. The final concentrations of Cr(VI) and sulfide were 40 μM and 800 μM, respectively, and the mineral loading was 5.0 g/L. The suspension was stirred by a magnetic Teflon bar. About 1 ml of suspension was periodically sampled by a 3 ml plastic syringe and immediately filtered through 0.22 μm membrane filter. Then, 0.50 ml filtrate was measured for Cr(VI) analysis.

The above experiments showed that Cr(VI) reduction was greatly enhanced in the system with illite, but depressed in the system with kaolinite and several other minerals. We, therefore, conducted more detailed experiments with illite and kaolinite in the pH range of 7.67 to 9.07, aiming to understand why the effects were so different. We hypothesized that the enhanced Cr(VI) reduction in the system with illlite could be due to the trace amount of iron associated with the mineral and/or surface catalysis. Several types of experiment were performed at pH 8.27 to evaluate whether ferrous iron from illite could alter the reaction rate. These included: (i) adding 5.0 μM soluble ferrous iron into the homogeneous system; (ii) introducing 0.10 mM phenanthroline into the illite suspension 30 min prior to initiation of the reaction; and (iii) adding 0.10 mM phenanthroline into the homogenous system as a control test. If ferrous iron was important for Cr(VI) reduction by sulfide, the reaction rate should increase with externally added Fe(II), but the effect be eliminated by phenanthroline because it could form strong complexes with Fe(II), hindering electron transfer to Cr(VI).

Elemental sulfur, the main product of sulfide oxidation by Cr(VI) in the anoxic system, is also known to accelerate Cr(VI) reduction in the later stage of the reaction, after initiated in the homogeneous system [21]. Experiments were therefore conducted to assess whether elemental sulfur had a similar impact on the reaction in the heterogeneous systems with illite and kaolinite. Stock solution of elemental sulfur was introduced into the reaction system at 50 μM final concentration level before the reaction was started.

### Cr(VI) and sulfide adsorption

The adsorption of Cr(VI) and sulfide on illite and kaolinite surfaces at pH 7.87 was assessed by monitoring the concentrations of Cr(VI) and sulfide in filtrates at defined time intervals. The initial concentrations of Cr(VI) and sulfide were 40 and 800 μM, respectively.

### Adsorbed and dissolved Fe(II)

The adsorbed and dissolved ferrous iron (Fe(II)) in illite suspension at pH 8.27 were monitored at defined time intervals. At each time point, 3.0 ml suspension was filtered through a 0.22 μm membrane, and then 1.0 ml of the filtrate was acidified with 1.0 ml of 0.1 M HCl and used for analysis of dissolved Fe(II) following the established ferrozine method [30,31]. To analyze the amount of sorbed Fe(II), the solids on the membrane were washed with 3 ml Mill-Q water to remove sulfide residual on the solids, and then the solids along with the membrane were transferred into 3.0 ml of 0.5 M HCl solution and mixed with a magnetic Teflon bar for 20 min for Fe(II) desorption. The suspension with desorbed Fe(II) was filtered with a 0.22 μm membrane filter and a 2.0 ml of the filtrate was mixed with 1.8 ml of 0.5 M NaOH to neutralize excess acid prior to determination of Fe(II) by ferrozine method.

### Analytical Methods

Cr(VI) concentration was determined by the diphenylcarbazide colorimetric method, using phosphoric buffer to control pH for the color development [26,32]. The absorbance was measured in a 1-cm cell at 540 nm on a spectrophotometer (Spectronic 20 Genesys, Spectronic Instruments). The method detection limit was approximately 0.05 μM and the precision was 5% rsd. Sulfide concentration in the stock solution was measured with the standard iodometric titration method [32]. Sulfide concentration during the reaction and the adsorption was monitored by the methylene blue colorimetric method with the absorbance measured at 664 nm [32,33].

Solution pH was constant during the reaction, as measured before the start and after the completion of the reaction by an Orion 420A pH meter after 2-point calibration. Dissolved oxygen was analyzed using the HACH dissolved oxygen test kit (HACH Company, Loveland, CO) in order to evaluate how completely the oxygen was removed by N_2 _purging. The dissolved oxygen in borate buffer after purging with N_2 _was less than the method detection limit of 6.3 μM. Dissolved oxygen was approximately 63 μM before purging with N_2_.

## Results

### Rates of Cr(VI) reduction by sulfide in the presence of various minerals

**Figure 1:**
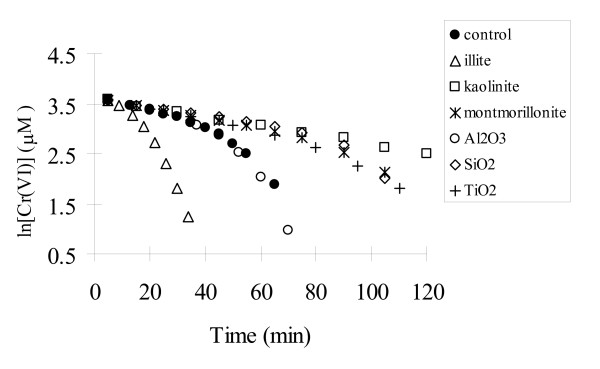
Effects of various minerals on Cr(VI) reduction by sulfide at pH 7.87. Initial concentrations were 40 μM for Cr(VI), 800 μM for sulfide, 5.00 g/L for solid mineral when present.

Three clay minerals (illite, kaolinite and montmorillonite) and three metal oxides (Al_2_O_3, _SiO_2 _and TiO_2_) were selected to assess the effect of minerals on the Cr(VI) reduction at pH 7.87. Initial concentrations of Cr(VI) and sulfide were 40 and 800 μM, respectively. Since sulfide concentration was at least 20 times as much as that of Cr(VI) during the reaction, a pseudo-first order condition was maintained with respect to [Cr(VI)]. Kinetic data, presented as the ln [Cr(VI)] v.s. time plots in Figure 1, however, did not follow the first order kinetics in most systems for the whole time period. In the control without any mineral, the line appears linear in the initial 40 minutes but curved downward, suggesting that the reaction was accelerated at the later stage of the reaction. This is consistent with the observation reported by Lan et al. [21]. The impact of minerals on Cr(VI) reduction could be assessed by comparing the rates between the systems containing a mineral and the control. As indicated by Figure 1, the tested minerals could be classified into three groups according to their effects on Cr(VI) reduction. Group 1 consisted of illite, which significantly accelerated Cr(VI) reduction. The time to complete the reaction was about 50% that of the control. The ln [Cr(VI)] v.s. time plot was not linear throughout the whole time period but curved downward, suggesting the presence of additional pathways that might have catalyzed the reaction. Group 2 mineral includes Al_2_O_3_, which has no observed effect when compared to the control. Also the ln [Cr(VI)] v.s. t plot was linear initially and then curved down. Group 3 minerals consisted of kaolinite, montmorillonite, SiO_2 _and TiO_2, _all of which inhibited Cr(VI) reduction by sulfide when compared to the control. Better linear trends in ln [Cr(VI)] v.s. time plots were observed through the reactions for montmorillonite, SiO_2_, and TiO_2_, although ln [Cr(VI)] v.s t plots curved down too at the later stage of the reaction. For the system with kaolinite, no late stage acceleration was observed during the whole experimental time period.

### Effects of pH

**Figure 2:**
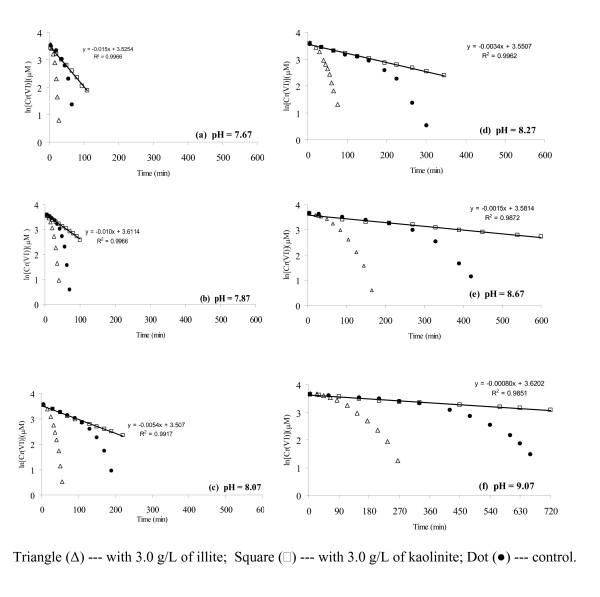
Plots of ln [Cr(VI)] vs. time show that the overall rate of Cr(VI) reduction is high with illite, low with kaolinite, and in the middle in the control without any mineral; and reaction rates decrease with increasing pH from pH 7.67 to pH 9.07. The initial concentrations of Cr(VI) and sulfide were 40 μM and 800 μM, respectively.

Cr(VI) reduction by sulfide in the systems with illite and kaolinite (3.0 g/L solid loading) was examined from pH 7.67 to 9.07 in comparison with the mineral-free control. From the ln [Cr(VI)] v.s. time plots in Figure 2(a–f), we observed that: (1) rates of Cr(VI) reduction by sulfide followed this order: illite > control > kaolinite; (2) in the illite suspension and in the control, the overall reaction was characterized by a slow initial reaction step, followed by a fast one; (3) in the kaolinite suspension, all plots of ln [Cr(VI) ] versus time were linear; and (4) reaction rates were increased significantly with decreasing pH from 9.07 to 7.67 for all systems, with and without minerals. When only the initial linear sections of ln [Cr(VI)] v.s. t plots were examined in the system with illite and in the control, prior to the points of downward curvature where about 35% to 50% (or about 15 to 20 μM) of the initial Cr(VI) was reduced, we found that the observed rate constants were almost the same as those in the kaolinite suspension.

### Uptake of Cr(VI) and Sulfide

Since the impact of minerals on Cr(VI) reduction could potentially be due to the sorption of reactants on the surfaces, we measured the sorption of both Cr(VI) and sulfide in separate batch systems at pH 7.87 as a function of time for 120 min. No detectable sorption of Cr(VI) was observed on the surfaces of illite or kaolinite minerals (3 g/L), as indicated by almost the same Cr(VI) concentration in the filtrate at the initial concentration of 40 μM. This result is not surprising because: (1) a much high concentration of borate buffer (0.10 M) may out-compete Cr(VI) (40 mM) for surface sites; and (2) the surfaces of illite (pH_pzc _= 4.5–5.0) and kaolinite (pH_pzc _= 3.5) were negatively charged at pH 7.87, discouraging sorption of negatively charged Cr_2_O_7_^2-^/CrO_4_^2-^. The observation was in agreement with results reported by Buerge ang Hug [25]. Also, no significant sorption of sulfide on kaolinite was observed at an initial sulfide concentration of 800 μM. However, approximately 13% of sulfide (i.e., 104 μM out of the original 800 μM) was lost in the system with 3.0 g/L of illite within 20 min, and stabilized afterward during the 120 min of testing period. The exact mechanism for the loss of sulfide was unclear; possible explanations included sorption on the mineral surfaces, precipitation as iron sulfide, and oxidation.

### Fe(II) sorbed onto illite

When illite exposed to the buffered aqueous solution at pH 8.27, we found the adsorbed amount of Fe(II) on illite (3 g/L) was approximately 7.0 μM. The amount did not increase with time during a testing period of 90 min in this control. In the presence of 800 μM sulfide, the adsorbed Fe(II) was increased by 4.0 μM in the initial period (< 30 min) over the control, and there was little change afterwards. This additional 4.0 μM of sorbed Fe(II) was small compared to the sulfide lost from the solution (104 mM) under comparable conditions. When 50 μM of elemental sulfur was also added, another 1.5 to 2.0 μM of sorbed Fe(II) was produced. Formation of polysulfides was expected when elemental sulfur and high concentration of sulfide were present. Polysulfides probably demonstrated higher reactivity towards reduction of structural Fe(III) in the illite, resulting in a slight increase in the sorbed amount of Fe(II) on the surfaces. In the experiments, there was no dissolved Fe(II) detected in the filtrate. This is expected because at this alkaline pH (8.27), Fe(II) could be strongly adsorbed onto the surface of illite or precipitated on mineral surfaces as insoluble species.

### Effect of Fe(II) on the reaction

**Figure 3:**
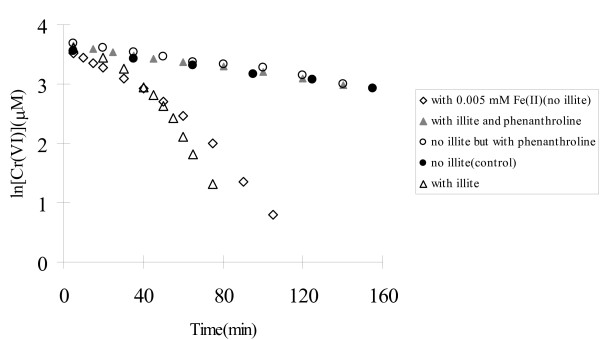
The presence of externally added ferrous iron and sorbed iron on illite (3.0 g/L) increases rates of Cr(VI) reduction by sulfide as tested at pH8.27. When phenanthroline, a strong Fe(II)-complexation agent, is added, the effect of illite with sorbed iron on the Cr(VI) reduction reaction was neutralized.

To diagnose whether Fe(II) associated with illite could have an impact on Cr(VI) reduction by sulfide, experiments were conducted in the homogenous system spiked with 5.0 μM ferrous chloride and the heterogeneous system with 3.0 g/L of illite. As shown in Figure 3, rates of Cr(VI) reduction in the systems with ferrous iron and illite were comparable, with a slow initial rate but being accelerated at the later stage of the reaction. In contrast, direct Cr(VI) reduction by sulfide in the control test was much slower and showed a linear ln [Cr(VI)] v.s. t plot within the whole experimental period. In the homogeneous system, the spiked Fe(II) apparently served as a catalyst for the reaction, i.e., Cr(VI) was reduced by Fe(II) to form Fe(III), which was then reduced by sulfide to regenerate Fe(II), enhancing the overall rate of Cr(VI) reduction. The fraction of Cr(VI) directly reduced by the initially added Fe(II) would not be significant because the spiked amount (5.0 μM) was much less than the total amount of Cr(VI) (40 μM) and it took three moles of Fe(II) to reduce one mole of Cr(VI). To understand if the sorbed iron on the illite surfaces played a similar role in the observed catalytic effect of illite, we conducted a set of experiments with illite in the presence of phenanthroline, a strong complexation agent for Fe(II). We hypothesized that if the sorbed iron on illite was responsible for the rate acceleration in the illite suspension, adding phenanthroline would decrease rate of Cr(VI) reduction because it formed strong complexes with Fe(II), known to inhibit Cr(VI) reduction in soils [34]. The effect of phenanthroline on Cr(VI) reduction in the homogeneous system was assessed in parallel as a control. The results, also presented in Figure 3, demonstrated that phenanthroline completely neutralized the effect of illite on the reduction of Cr(VI), although it had no effect on the reaction in the homogeneous system. In the three systems: (1) illite and phenanthroline, (2) control (homogeneous) and (3) homogeneous system with phenanthroline, all ln [Cr(VI)] v.s. time plots were linear with near identical slopes (rate constants), indicating the reaction order with respect to Cr(VI) was first order during the 160 min of experimental time period. These results suggested that the sorbed Fe(II) on illite must have catalyzed Cr(VI) reduction, and when it was coordinated with phenanthroline, the catalytic reactivity was inhibited.

### Effect of externally-added elemental sulfur

**Figure 4:**
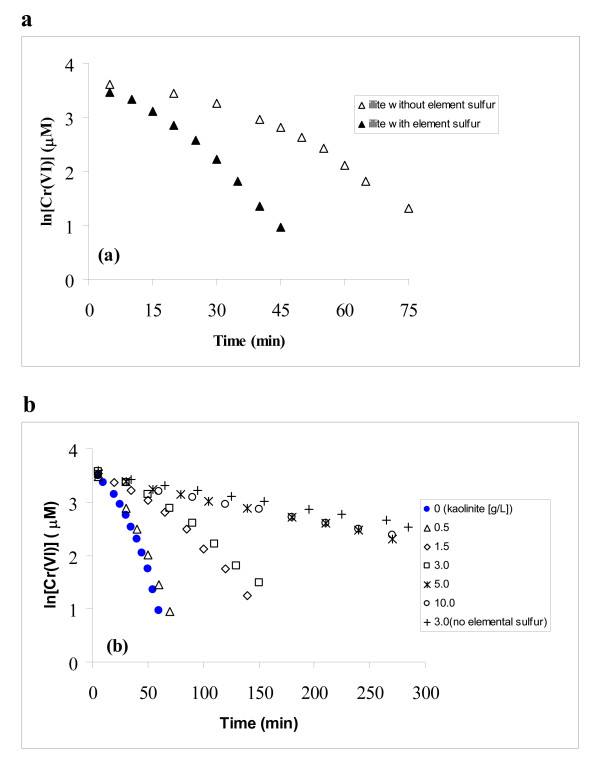
Effect of externally-added elemental sulfur on the reduction of Cr(VI) by sulfide at pH 8.27. (a) in 3.0 g/L illite suspension, (b) in kaolinite suspensions with various solid loadings.

As shown in Figure 4a, 50 μM of externally-added elemental sulfur could accelerate the reduction of Cr(VI) by sulfide in the illite suspension (3.0 g/L): the time to near complete reduction of 40 μM Cr(VI) was shortened from 75 min to 45 min by adding elemental sulfur.

For kaolinite suspension, the effect of elemental sulfur was examined in a set of tests with kaolinite loadings increasing from 0 to 10.0 g/L (Figure 4b). Significant rate enhancement by elemental sulfur was observed at low kaolinite loadings from 0.0 – 3.0 g/L. For example, with 3.0 g/L of kaolinite, the time required to reduce 40 μM of Cr (VI) was approximately 150 min, which was much shorter than the rate without elemental sulfur where only 70% of the Cr(VI) was reduced within 300 min. However, the effect of elemental sulfur was less pronounced when the amount of kaolinite was increased. At a 0.50 g/L level, the effect of kaolinite was barely detectable; and when kaolinite was at or higher than 5.0 g/L, the plots of ln [Cr(VI)] v.s. time were almost the same as for the system without externally-added elemental sulfur. The observed rate constants in the two tests with 50 μM of added elemental sulfur but also the highest kaolinite concentrations (*k*_obs _= 4.1 × 10^-3 ^min ^-1^) were very close to that in the suspension without added elemental sulfur (*k*_obs _= 3.5 × 10^-3 ^min ^-1^), indicating that the effect of externally-added elemental sulfur was neutralized by the high concentration of kaolinite.

### Effect of sulfide concentration

**Figure 5:**
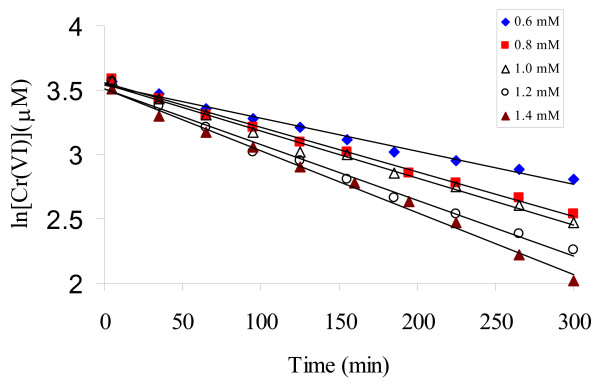
Effect of sulfide concentration on the reduction of Cr(VI) by sulfide at pH 8.27 in 3.0 g/L kaolinite suspension.

Increasing sulfide concentration from 600 to 1400 μM resulted in accelerated Cr(VI) reduction in the presence of kaolinite (3.0 g/L) at pH 8.27, as shown in Fig. 5. The ln [Cr(VI)] v.s. t plots were all linear under the experimental conditions with r^2 ^> 0.99 for all experiments.

## Discussion

Previous studies [20,21] have shown that the stoichiometry for the aqueous phase Cr(VI) reduction by sulfide is: 2CrO_4_^2- ^+ 3HS^- ^+ 7H^+ ^= 2Cr(OH)_3_(s) + 3S(s) + 2H_2_O, under anoxic conditions. The presence of minerals as examined in the study is not expected to change the stoichiometry, but only the kinetics.

The examined minerals fall into three groups based on their effects on the rate of Cr(VI) reduction by sulfide: illite in Group 1 exhibits remarkable catalysis; Al_2_O_3 _in Group 2 shows no significantly effect in comparison with the homogeneous control; and TiO_2_, SiO_2_, kaolinite and montmorillonite, all in Group 3, inhibit Cr(VI) reduction to various degrees. Different effects demonstrated by these minerals can not be explained by their differences in specific surface area (SSA) as listed in Table 1. For example, illite has the lowest SSA among all tested minerals but can accelerate the reaction most dramatically. Other minerals with larger SSA such as kaolinite, montmorillonite, Al_2_O_3 _and SiO_2 _also show different effects on the Cr(VI) reduction reaction.

By comparing Cr(VI) concentration in the filtrate of illite and kaolinite suspensions with its initial concentration, we find insignificant sorption of Cr(VI) for either of these two minerals. In addition, the reduction rates of Cr(VI) by sulfide in these two suspensions are quite different. It seems significant adsorption of Cr(VI) may not be required for Cr(VI) reduction. As for sulfide, while a maximal 13% of initial sulfide is lost in the system with illite, probably due to sorption, kinetic of Cr(VI) reduction in the illite suspension with phenanthroline is almost identical to those in kaolinite suspension and in homogeneous system (Fig. 3 and 4b). Our unreported data show that phenanthroline does not influence sulfide adsorption on goethite, so it may not affect sulfide sorption on illite either. Therefore, the sorption of sulfide is also unlikely to be a key factor that could result in catalysis of Cr(VI) in the illite suspension. Instead, Fe(II)/Fe(III), likely coordinated with hydroxyl group in the tested pH range of 7.67 to 9.07, must be involved in accelerating the reduction of Cr(VI) by sulfide in illite suspension. This agrees with the observations that (1) phenanthroline can block Cr(VI) reduction by sorbed Fe(II) through formation of very stable complex and (2) soluble ferrous iron addition to homogeneous systems greatly enhances rate of Cr(VI) reduction.

We have previously reported that the overall kinetics of Cr(VI) reduction by sulfide in the homogenous system can be expressed by the following empirical equation[21]:

−d[Cr(VI)]dt=k1[Cr(VI)][HS-]0.63+k2[Cr(VI)][≡S-SH]0.57
 MathType@MTEF@5@5@+=feaafiart1ev1aaatCvAUfKttLearuWrP9MDH5MBPbIqV92AaeXatLxBI9gBamXvP5wqSXMqHnxAJn0BKvguHDwzZbqegyvzYrwyUfgarqqtubsr4rNCHbGeaGqiA8vkIkVAFgIELiFeLkFeLk=iY=Hhbbf9v8qqaqFr0xc9pk0xbba9q8WqFfeaY=biLkVcLq=JHqVepeea0=as0db9vqpepesP0xe9Fve9Fve9GapdbaqaaeGacaGaaiaabeqaamqadiabaaGcbaGaeyOeI0YaaSaaaeaacqWGKbazcqGGBbWwcqqGdbWqcqqGYbGCcqGGOaakcqGGwbGvcqGGjbqscqGGPaqkcyGGDbqxaeaacqWGKbazcqWG0baDaaGaeyypa0Jaem4AaS2aaSbaaSqaaiabigdaXaqabaGccqGGBbWwcqqGdbWqcqqGYbGCcqGGOaakcqGGwbGvcqGGjbqscqGGPaqkcyGGDbqxcqGGBbWwcqqGibascqqGtbWudaahaaWcbeqaaiabc2caTaaakiGbc2faDnaaCaaaleqabaGaeGimaaJaeiOla4IaeGOnayJaeG4mamdaaOGaey4kaSIaem4AaS2aaSbaaSqaaiabikdaYaqabaGccqGGBbWwcqqGdbWqcqqGYbGCcqGGOaakcqGGwbGvcqGGjbqscqGGPaqkcyGGDbqxcqGGBbWwcqGHHjIUcqqGtbWucqGGTaqlcqGGtbWucqGGibascyGGDbqxdaahaaWcbeqaaiabicdaWiabc6caUiabiwda1iabiEda3aaaaaa@7A46@

Where the first term represents the reaction between aqueous Cr(VI) and sulfide, and the second term represents Cr(VI) reduction by polysulfide/sorbed sulfide on elemental sulfur nanoparticles. Considering that Fe(II) is involved in Cr(VI) reduction in the heterogeneous system, such as in illite suspension, and the reaction is first order with respect to both Cr(VI) and Fe(II) as reported by Buerge et al. [8,25], the empirical equation (1) could be modified to:

−d[Cr(VI)]dt=k1[Cr(VI)][HS-]0.63+k2[Cr(VI)][≡S-SH]0.57+k3[Cr(VI)][Fe(II)]
 MathType@MTEF@5@5@+=feaafiart1ev1aaatCvAUfKttLearuWrP9MDH5MBPbIqV92AaeXatLxBI9gBamXvP5wqSXMqHnxAJn0BKvguHDwzZbqegyvzYrwyUfgarqqtubsr4rNCHbGeaGqiA8vkIkVAFgIELiFeLkFeLk=iY=Hhbbf9v8qqaqFr0xc9pk0xbba9q8WqFfeaY=biLkVcLq=JHqVepeea0=as0db9vqpepesP0xe9Fve9Fve9GapdbaqaaeGacaGaaiaabeqaamqadiabaaGcbaGaeyOeI0YaaSaaaeaacqWGKbazcqGGBbWwcqqGdbWqcqqGYbGCcqGGOaakcqGGwbGvcqGGjbqscqGGPaqkcyGGDbqxaeaacqWGKbazcqWG0baDaaGaeyypa0Jaem4AaS2aaSbaaSqaaiabigdaXaqabaGccqGGBbWwcqqGdbWqcqqGYbGCcqGGOaakcqGGwbGvcqGGjbqscqGGPaqkcyGGDbqxcqGGBbWwcqqGibascqqGtbWudaahaaWcbeqaaiabc2caTaaakiGbc2faDnaaCaaaleqabaGaeGimaaJaeiOla4IaeGOnayJaeG4mamdaaOGaey4kaSIaem4AaS2aaSbaaSqaaiabikdaYaqabaGccqGGBbWwcqqGdbWqcqqGYbGCcqGGOaakcqGGwbGvcqGGjbqscqGGPaqkcyGGDbqxcqGGBbWwcqGHHjIUcqqGtbWucqGGTaqlcqGGtbWucqGGibascyGGDbqxdaahaaWcbeqaaiabicdaWiabc6caUiabiwda1iabiEda3aaakiabgUcaRiabdUgaRnaaBaaaleaacqaIZaWmaeqaaOGaei4waSLaee4qamKaeeOCaiNaeiikaGIaeiOvayLaeiysaKKaeiykaKIagiyxa0Laei4waSLaeeOrayKaeeyzauMaeiikaGIaeiysaKKaeiysaKKaeiykaKIagiyxa0faaa@8F81@

Since the concentrations of ≡S-SH and Fe(II) increase with time in the system with illite, rates of Cr(VI) reaction could be accelerated, which explains why the reaction does not follow a first order kinetics throughout the whole experimental duration, even though the sulfide concentration is maintained constant. It should be pointed that in the illite suspension with pH from 7.67 to 9.07 investigated in this study, FeS is likely the main species of Fe(II) as suggested by Patterson and Fendorf [24] and Morse et al. [35]. Nevertheless, freshly formed FeS can also quickly reacts with Cr(VI)[24], so delineation of exact Fe(II) species is not essential to understand Cr(VI) reduction by sulfide in our systems.

Considering that phenanthroline will block Fe(II) as a reductant and in the initial stage of the reaction, the effect of elemental sulfur product on the overall reaction is not important as reported by Lan et al. [21]. Equation (2) can thus be written as:

-*d*[Cr(VI)]/*dt *= *k*_obs _[Cr(VI)]

where *k*_obs _= *k*_1 _[HS^-^]^0.63^. The overall reaction now becomes pseudo first order with respect to Cr(VI) under this condition. This agrees with our experimental observations that the plot of ln [Cr(VI)] v.s. time is linear (with r^2 ^= 0.996) within 150 min of reaction, when approximately 50% of initial Cr(VI) is reduced (Fig. 3). The rate constant *k*_obs _was 0.0047 min^-1^, very close to the *k*_obs _values of 0.0040 min^-1 ^(r^2 ^= 0.992) and 0.0048 min^-1 ^(r^2 ^= 0.986) obtained from the homogeneous systems without and with phenanthroline, respectively.

In this study, total amount of ferrous iron is low: with 5 μM spiked into the homogeneous system and 7.0 μM of Fe(II) detected in the presence of illite. According to a Cr(VI)/Fe(II) molar ratio of 1:3 for the reaction, only about 1–2 μM of Cr(VI) could be consumed by Fe(II), which is insignificant when compared to the initial Cr(VI) concentration of 40 μM. However, rates of Cr(VI) reduction by sulfide were significantly different with and without iron. It is therefore likely that Fe(II)/Fe(III) serves as an electron shuttle, mediating the electron transfer between Cr(VI) and sulfide as illustrated below:



This interpretation is consistent with our experiments showing that Cr(VI) reduction by Fe(II) is much faster than the reduction of Cr(VI) by sulfide at pH 7 – 9, as well as the results reported in the literature[36].

Kaolinite is one of the minerals that inhibit Cr(VI) reduction by sulfide. In the presence of kaolinite, the kinetic results (*k*_obs_) obtained at pH ranging from 7.67 to 9.07 are all close to those in the homogeneous system (Fig. 2a–f) at the initial stage of the reaction, when the elemental sulfur is expected to exert no effect on the reaction. It is likely that the elemental sulfur produced from the reaction is sequestered by kaolinite, so the catalytic effect of elemental sulfur is eliminated. Under this circumstance, the second term in Equation (1) is negligible and a pseudo-first order dependence on Cr(VI) should be observed. This is exactly what we have measured in the experiments (Figure 5).

When the effect of pH is examined on Cr(VI) reduction in the kaolinite suspension, a generic rate equation can be written as:

-*d*[Cr(VI)]/*dt *= *k*[Cr(VI)][HS^-^]^a^[H^+^]^b ^

**Figure 6:**
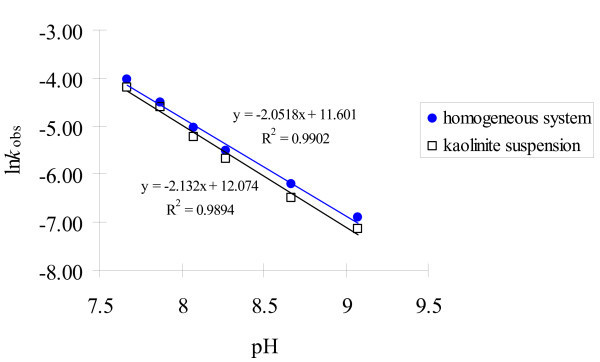
ln*k*_obs _as a function of pH in 3 g/L kaolinite suspension and homogeneous system. The *k*_obs _in the homogeneous system is for the initial stage of the reaction only, when approximately 35 to 50% of initial Cr(VI) was consumed.

If sulfide concentration and solution pH are kept constant, Equation (5) is simplified to Equation (3) again, with *k*_obs _= *k *[HS^-^]^a ^[H^+^]^b^. Using the experimental results in Figure 2 under various pH values, we could evaluate the pH dependence of the reaction in the kaolinite system by the relationship: ln *k*_obs _= ln*k *[HS^-^]^a ^-b pH= *K *– b pH. As shown by Figure 6, the ln *k*_obs _v.s. pH plots for the kaolinite suspension and the initial stage of the homogeneous reaction are linear, with the slopes of -2.13 and -2.05, respectively. The result suggests that the reaction order with respect to H^+ ^is almost 2, higher than the 1 reported by Pettine et al. [19].

**Figure 7:**
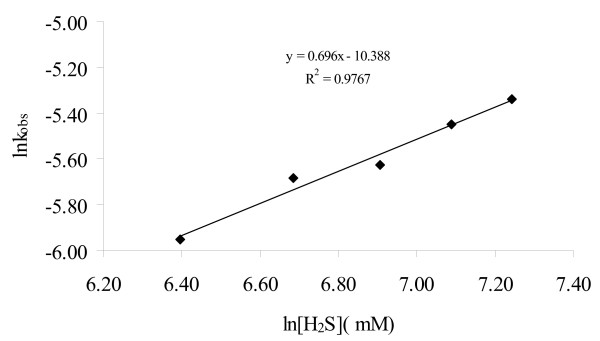
Values of ln *k*_obs _as a function of ln[HS^-^]_total _at pH 8.27 and in 3.0 g/L kaolinite suspension.

At constant pH of 8.27, the rate constant (*k*_obs_) increased with increasing sulfide concentration, with a slope of 0.70 in the ln *k*_obs _versus ln [H_2_S] plot (Figure 7). A fractional reaction order of 0.70 with respect to total sulfide in the kaolinite suspension is close to the reaction of of 0.63 in the homogeneous system [21], but is lower than the reaction orders reported by Pettine et al. [6,19] and Kim et al. [20].

The effect of externally added elemental sulfur (50 μM) on Cr(VI) reduction in the kaolinite suspension is strongly dependent on kaolinite loading. Cr(VI) reduction is enhanced by elemental sulfur at kaolinite loading less than 3.0 g/L (see Fig. 4b). At 0.5 g/L kaolinite, the rate is almost the same as that in the control without kaolinite. When kaolinite concentration is increased to 5.0 g/L and higher, however, the catalytic effect of elemental sulfur disappears. It seems the externally added elemental sulfur is also sequestered by kaolinite, eliminating its catalytic effect. From Figure 1, we know that 3.0 g/L of kaolinite can eliminate the catalytic effect of elemental sulfur produced from reaction with 40 μM Cr(VI), which is 60 μM. When an additional 50 μM of elemental sulfur is added externally, 5.0 g/L or more kaolinite is needed to eliminate the effect of elemental sulfur, which is consistent with the observation shown in Fig. 4b.

No detailed experiment was conducted to examine the inhibitive effect of montmorillonite, TiO_2 _and SiO_2 _on the reduction of Cr(VI) by sulfide. It is expected that elemental sulfur is similarly sequestered by montmorillonite, SiO_2_, and Al_2_O_3_, losing its catalytic reactivity.

## References

[B1] Deng B (1995). Chromium(VI) Reduction by Naturally-Occurring Organic Compounds: Direct and Surface-Catalyzed Reactions.

[B2] James BR (1996). The challenge of remediating chromium-contaminated soil. Environmental Science and Technology.

[B3] Blowes DW, Ptacek CJ, Jambor JL (1997). In-Situ Remediation of Chromate Contaminated Groundwater Using Permeable Reactive Walls. Environmental Science & Technology.

[B4] Eary LE, Rai D (1988). Chromate Removal from Aqueous Wastes by Reduction with Ferrous Ion. Environmental Science & Technology.

[B5] Sedlak DL, Chan PG (1997). Reduction of Hexavalent Chromium (VI) by Ferrous Iron. Geochim Cosmochim Acta.

[B6] Pettine M, D'Ottone L, Campanella L, Millero FJ, Passino R (1998). The Reduction of Chromium (VI) by Iron (II) in Aqueous Solution. Geochimica et Cosmochimica Acta.

[B7] Buerge IJ, Hug SJ (1997). Kinetics and pH Dependence of Chromium (VI) Reduction by Iron (II). Environmental Science & Technology.

[B8] Buerge IJ, Hug SJ (1998). Influence of Organic Ligands on Chromium (VI) Reduction by Iron (II). Environmental Science & Technology.

[B9] Seaman JC, Bertsch PM, Schwallie L (1999). In Situ Cr (VI) Reduction within Coarse-Textured, Oxide-Coated Soil and Aquifer Systems Using Fe (II) Solution. Environmental Science & Technology.

[B10] Fruchter J (2002). *In Situ *Treatment of Chromium-contaminated Groundwater. Environmental Science & Technology.

[B11] Bond D, Fendorf S (2003). Kinetics and Structural Constraints of Chromate Reduction by Green Rusts. Environmental Science & Technology.

[B12] James B, Bartlett RJ (1983). Behavior of Chromium in Soils. VI. Interactions Between Oxidation-Reduction and Organic Complexation. J Environ Qual.

[B13] Wittbrodt PR, Palmer CD (1995). Reduction of Cr (VI) in the Presence of Excess of Soil Fulvic Acid. Environmental Science & Technology.

[B14] Goodgame D-L, Hayman PB (1984). Formation of Water-soluble Chromium(V) by the Interaction of Humic Acid and the Carcinogen Chromium(VI). Inorganica Chimica Acta.

[B15] Thornton EC, Amonette JE (1997). Gas Treatment of Cr(VI)-contaminated Sediment Samples from the North 60's Pits of the Chemical Waste Landfill; PNNL-11634.

[B16] Thornton EC, Amonette JE (1999). Hydrogen Sulfide Gas Treatment of Cr (VI)-Contaminated Sediment Samples from a Plating-Waste Disposal Site. Implication for in-Situ Remediation. Environmental Science & Technology.

[B17] ASME (1999). Technical Peer Review Report in Assessment of Technologies Supported by the Office of Science and Technology Department of Energy.

[B18] Cantrell KJ, Yabusaki SB, Engelhard MH, Mitroshkov AV, Thornton EC (2003). Oxidation of H_2_S by Iron Oxides in Unsaturated Conditions. Environ Sci Technology.

[B19] Pettine M, Millero FJ, Passino R (1994). Reduction of Chromium (VI) with Hydrogen Sulfide in NaCl Media. Marine Chemistry.

[B20] Kim C, Zhou Q, Deng B, Thornton EC, Xu H (2001). Chromium (VI) Reduction by Hydrogen Sulfide in Aqueous Media: Stoichiometry and Kinetics. Environmental Science & Technology.

[B21] Lan Y, Deng B, Kim C, Thornton EC, Xu H (2005). Catalysis of Elemental Sulfur Nanoparticles on Chromium (VI) Reduction by Sulfide under Anaerobic Conditions. Environmental Science & Technology.

[B22] Hua B, Deng B (2003). Influences of Water Vapor on Cr(VI) Reduction by Gaseous Hydrogen Sulfide. Environmental Science & Technology.

[B23] Eary LE, Rai D (1989). Kinetics of Chromate Reduction by Ferrous Ions Derived From Hematite and Biotite at 25°C. Am J Sci.

[B24] Patterson RR, Fendorf S (1997). Reduction of Hexavalent Chromium by Amorphous Iron Sulfide. Environmental Science & Technology.

[B25] Buerge IJ, Hug SJ (1999). Influence of Mineral Surfaces on Chromium (VI) Reduction by Iron (II). Environmental Science & Technology.

[B26] Deng B, Stone AT (1996). Surface-Catalyzed Chromium (VI) Reduction: The TiO_2_-Mandelic Acid System. Environmental Science & Technology.

[B27] Deng B, Stone AT (1996). Surface-Catalyzed Chromium(VI) Reduction: Reactivity Comparisons among Different Organic Reductants and Different Catalytic Surfaces. Environmental Science & Technology.

[B28] Stumn W, Morgan JJ (1996). Aquatic Chemistry.

[B29] Zhachara JM, Davis JA, Liu C, McKinley JP, Qafoku N, Wellman DM, Yabusaki SB (2005). Uranium Geochemistry in Vadose Zone and Aquifer Sediments from the 300 Area Uranium Plum.

[B30] Amonette JE, Workman AJ, Kennedy DW, Fruchter JS, Gorby YA (2000). Dechlorination of Carbon Tetrachloride by Fe(II) Associated with Goethithe. Environmental Science & Technology.

[B31] Lovley DR, Philips E-P (1986). Availability of Trivalent Iron for Microbial Reduction in Bottom Sediments of the Freshwater Tidal Potomac River. Appl And Environ Microbiol.

[B32] APHA; AWWA; WPCF (1998). Standard Methods for the Examination of Water and Wastewater.

[B33] Allen HE, Fu G, Deng B (1993). Analysis of Acid Volatile Sulfide (AVS) and Simultaneously Extracted Metals (SEM) for the Estimation of Potential Toxicity in Aquatic Sediments. Environ Toxicol Chem.

[B34] Anderson LD, Kent DB, Davis JA (1994). Batch Experiments Characterizing the Reduction of Cr(VI) Using Suboxic Material from a Mildly Reducing Sand and Gravel Aquifer. Environmental Science & Technology.

[B35] Morse JW, Millero FJ, Cornwell JC, Rickard D (1987). The Chemistry of the Hydrogen Sulfide and Iron Sulfide Systems in Natural Waters. Earth Sci Rev.

[B36] Lan Y, Yang J, Deng B (2006). Catalysis of Dissolved and Adsorbed Iron in Soil Suspension for Chromium(VI) Reduction by Sulfide. Pedosphere.

